# Exosomes derived from three-dimensional cultured human umbilical cord mesenchymal stem cells ameliorate pulmonary fibrosis in a mouse silicosis model

**DOI:** 10.1186/s13287-020-02023-9

**Published:** 2020-11-25

**Authors:** Chunjie Xu, Jing Zhao, Qiuyue Li, Lin Hou, Yan Wang, Siling Li, Fuyang Jiang, Zhonghui Zhu, Lin Tian

**Affiliations:** 1 Department of Occupational and Environmental Health, School of Public Health, Capital Medical University, No. 10, Xitoutiao Youanmen Street, Beijing, 100069 China; 2 Beijing Key Laboratory of Environmental Toxicology, Capital Medical University, Beijing, 100069 China

**Keywords:** Silicosis, Pulmonary fibrosis, 3D, hucMSC-Exos

## Abstract

**Background:**

Silicosis is an occupational respiratory disease caused by long-term excessive silica inhalation, which is most commonly encountered in industrial settings. Unfortunately, there is no effective therapy to delay and cure the progress of silicosis. In the recent years, stem cell therapy has emerged as an attractive tool against pulmonary fibrosis (PF) owing to its unique biological characteristics. However, the direct use of stem cells remains limitation by many risk factors for therapeutic purposes. The exclusive utility of exosomes secreted from stem cells, rather than cells, has been considered a promising alternative to overcome the limitations of cell-based therapy while maintaining its advantages.

**Methods and results:**

In this study, we first employed a three-dimensional (3D) dynamic system to culture human umbilical cord mesenchymal stem cell (hucMSC) spheroids in a microcarrier suspension to yield exosomes from serum-free media. Experimental silicosis was induced in C57BL/6J mice by intratracheal instillation of a silica suspension, with/without exosomes derived from hucMSC (hucMSC-Exos), injection via the tail vein afterwards. The results showed that the gene expression of collagen I (COL1A1) and fibronectin (FN) was upregulated in the silica group as compared to that in the control group; however, this change decreased with hucMSC-Exo treatment. The value of FEV0.1 decreased in the silica group as compared to that in the control group, and this change diminished with hucMSC-Exo treatment. These findings suggested that hucMSC-Exos could inhibit silica-induced PF and regulate pulmonary function. We also performed in vitro experiments to confirm these findings; the results revealed that hucMSC-Exos decreased collagen deposition in NIH-3T3 cells exposed to silica.

**Conclusions:**

Taken together, these studies support a potential role for hucMSC-Exos in ameliorating pulmonary fibrosis and provide new evidence for improving clinical treatment induced by silica.

**Supplementary information:**

The online version contains supplementary material available at 10.1186/s13287-020-02023-9.

## Introduction

Silicosis is one of the main occupational respiratory diseases caused by inhalation of silica excessively, which is most commonly encountered in industrial settings [1]. It presents as a diffuse interstitial disease, and it is characterized by silicotic nodules and progressive lung fibrosis [2]. According to previous studies, multiple factors, such as impaired alveolar epithelial cells, macrophage activation, fibroblast proliferation, and collagen production, have been implicated in the pathogenesis of pulmonary fibrosis [3, 4]. To a certain extent, the incidence of silicosis has been controlled through the efforts of many countries. However, it still remains a serious public health concern, especially in certain developing nations. Over the past 30 years, China has recorded more than 600,000 cases, the highest silicosis burden worldwide. Furthermore, the number of new cases is still increasing [5]. Unfortunately, there is no effective therapy to postpone the progress of silicosis. Thus, it is necessary to explore the molecular mechanisms of silicosis in depth to develop therapeutic avenues.

At present, stem cell therapy offers hope for the treatment of pulmonary fibrosis owing to its unique biological characteristics, such as multidirectional differentiation, immune regulation, and paracrine effects [6–8]. However, the direct use of stem cells remains limitation by many risk factors for therapeutic purposes, such as thrombosis, and undesired immune responses [9]. Exosomes are the key mediators of paracrine cells because they transfer genetic material and proteins to target cells. Exogenous exosomal molecules can regulate target gene or protein expression and receptor cell functions [10].

Exosomes are small membrane particles with a size of 40–150 nm, which play crucial roles in intercellular communication by delivering miRNAs, mRNAs, and proteins to recipient cells [11]. Studies [12, 13] have reported that the exosomes secreted by hucMSCs can promote wound closure in burn-wound animal models. Li et al. [12] found that 3 weeks after receiving hucMSC-Exo injection, mice with CCl4-induced liver injury showed a reduction in liver fibrosis.

As a large quantity of exosomes secreted from hucMSC-Exos are sought after for therapeutic use, it is important to generate hucMSC-Exos at a large scale while maintaining their advantageous characteristics. To resolve this issue, several studies have developed dynamic culturing methods for 3D mesenchymal stem cells (MSCs) to yield exosomes rather than traditional culturing methods for 2D MSCs [14, 15]. Nevertheless, to date, few studies have directly utilized hucMSC-Exos for the treatment of silicosis. Therefore, our study aims to isolate exosomes from hucMSCs based on 3D cultures and explore the effects of anti-fibrosis measures.

## Material and methods

### Cell culture

3D FloTrix miniSpin bioreactor (Beijing CytoNiche Biotechnology Co. Ltd., Beijing, China) is used for scalable microcarrier-based 3D dynamic culture of hucMSCs, and it mainly includes a miniSpin agitator and spinner flasks of three volumes. hucMSCs were seeded to a density of 5 × 10^4^ cells/mL in basal medium supplemented with 4% supplement medium; the speed of the miniSpin agitator was set to 50 rpm/min, and cells were cultured at 37 °C in a 5% CO_2_ incubator. During the cell culture, cell viability was determined using calcein AM and propidium iodide (PI) kit (Wako, Japan) according to the manufacturer’s instruction.

RAW264.7 cells (peritoneal macrophages of mice) could be activated and release some inflammatory mediators. These substances may impair pulmonary tissues and stimulate fibroblast (NIH-3T3 cells) proliferation and differentiation into myofibroblasts.

RAW264.7 cells were treated with 50 μg/mL silica for 24 h, and the supernatant was collected. NIH-3T3 cells (lung fibroblasts of mice) were maintained with the original silica supernatant for 24 h and harvested for future experiments. See additional detail in online supplement.

### Isolation and identification of hucMSC-Exos

Serum-free medium of the 3D culture was centrifuged at 3000*g* for 15 min to remove dead cells and cellular debris. Exoquick exosome precipitation solution (System Biosciences, Palo Alto, USA) was added to the ultrafiltrate, mixing thoroughly by inversion. Exosomes were stored at − 80 °C or used in downstream experiments. See additional detail in online supplement.

### Transmission electron microscopy

hucMSC-Exos were resuspended with PBS at a concentration of 100 μg/mL. Then, the mixture was applied to the copper grid and fixed with 2% phosphotungstic acid negatively. A transmission electron image was acquired using an HT-7700 transmission electron microscope (Hitachi, Tokyo, Japan).

### Nanoparticle tracking analysis

In order to quantify hucMSC-Exos and size distribution, NanoSight instrument (ZetaVIEW, Germany) and ZetaView 8.04.02 software were used for nanoparticle tracking analysis (NTA). For NTA, 10 μL hucMSC-Exos was diluted to 20,000 times gradually and automatically calculating the size of at least 10,000 particles. The PBS was evaluated before the experiment to ensure that it was free of particles.

### In vitro tracking

hucMSC-Exos were fluorescently labeled with 1,1′-dioctadecyl-3,3,3′,3′-tetramethylindocarbocyanine perchlorate (Dil, Beyotime Biotechnology, Shanghai, China). The labeled exosomes were isolated through Exoquick exosome precipitation solution (System Biosciences, Palo Alto, USA) to remove excess dye. Mixtures were co-cultured with NIH-3T3 cells in DMEM for 4 h at 37 °C. Then, the cells were washed thrice with PBS to remove excess dye, co-cultured with Hoechst dye (Beyotime Biotechnology, Shanghai, China) at 37 °C for 1 h, and then observed under a fluorescence microscope.

### Animal model of silicosis and ethics statement

A total of 60 C57BL/6J mice were randomly divided into three groups: the control (*n* = 20), silica (*n* = 20), and silica + hucMSC-Exos (*n* = 20). This study was approved by the Laboratory Animal Care and Use Committee at Capital Medical University (AEEI-2018-223) and abided by the principle of reduction, replacement, and refinement described in the National Institute of Health Guide for the Care and Use of Laboratory Animals. See additional detail in online supplement.

### In vivo tracking

Mice were intravenously injected with hucMSC-Exo labeled with fluorescent 1,1′-dioctadecyl-3,3,3′,3′-tetramethylindotricarbocyanine iodide (DiR, Life Technologies, Carlsbad, CA, USA). The Carestream FX Pro imaging system (Bruker BioSpin MRI GmbH, Ettlingen, Germany) was used to capture the fluorescent signal at different time points (1, 6, 24, 48, 72, and 96 h) in the body. See additional detail in online supplement.

### Lung function measurements

The flexiVent FX system (SCIREQ, Inc., Montreal, Canada) was used for respiratory function measurement. The system was equipped with an FX2 module and operated using the Flexi Ware v8.0 software. See additional detail in online supplement.

### Western blotting

The samples of hucMSC-Exos were subjected to SDS-PAGE on 10% gels. The following primary antibodies were used: CD81 (1:1000, ab33697) and TSG101 (1:1000, ab125011) were purchased from Abcam (Abcam, USA) and CD63 (1:1000, 25682-1-AP) was procured from Proteintech (Proteintech, USA). The protein of NIH-3T3 cells was resolved on 8% SDS polyacrylamide gels. The primary antibodies, COL1A1 (1:100, sc293182) and FN (1:200, sc8422), were purchased from Santa Cruz (Santa Cruz, USA), and GAPDH (1:1000, 2118s) was obtained from Cell Signaling Technology (Cell Signaling Technology, USA). ECL detection reagent (Absin, Shanghai, China) was used to detect the signals, and the signals were imaged by Tanon-5200 system (Beijing Yuan Ping Hao Biotech, China). The quantification was calculated by ImageJ software to analyze the intensity of the gray scale images. See additional detail in online supplement.

### RNA isolation and reverse transcription quantitative PCR (RT-qPCR)

The levels of gene expression were calculated by normalizing to the glyceraldehyde-3-phosphate dehydrogenase level. The COL1A1 sense sequence was 5′-GCTCCTCTTAGGGGCCACT-3′, and the antisense sequence was 5′-CCACGTCTCACCATTGGGG-3′. The FN sense sequence was 5′-CTATAGGATTGGAGACACGTGG-3′, and the antisense sequence was 5′-CTGAAGCACTTTGTAGAGCATG-3′. See additional detail in online supplement.

### Histopathology

The left lung of mice was inflated overnight with 10% formalin. The organs were embedded in paraffin and cut into 5-μm-thick slices. The sections were observed using 3D histological technologies (Pannoramic Scan, Japan) with × 200 or × 400 magnification.

### Pulmonary hydroxyproline assay

Pulmonary hydroxyproline (HYP) was detected using a conventional hydroxyproline colorimetric assay kit (Nanjing, China). Protein levels in the lung tissue were detected at an absorbance of 550 nm based on the manufacturer’s instruction, and all data were expressed in milligrams per gram of protein.

### Confocal immunofluorescence

The sample of NIH-3T3 cells was fixed with 0.1% Triton X-100 and blocked with 3% BSA, and then incubated with primary antibodies against COL1A1 (1:100, sc293182) and FN (1:100, sc8422) overnight at 4 °C. The cell climbing piece was incubated with secondary antibodies conjugated to fluorescein isothiocyanate at room temperature for 1 h, which was counterstained with DAPI subsequently. The images were captured with a Leica TCS SP8 STED confocal microscope (Leica Microsystems, Germany).

### Statistical analysis

Statistical analysis was performed by SPSS software version 19. All the data were presented as mean ± standard deviation (SD). Student’s *t* test was used for comparisons of two groups, and one-way analysis of variance (ANOVA) was used for multiple-group comparisons. Imagines were performed using GraphPad Prism software. Differences were considered to be statistically significant when *P* < 0.05.

## Results

### 3D cultivation of hucMSCs

**Fig. 1:**
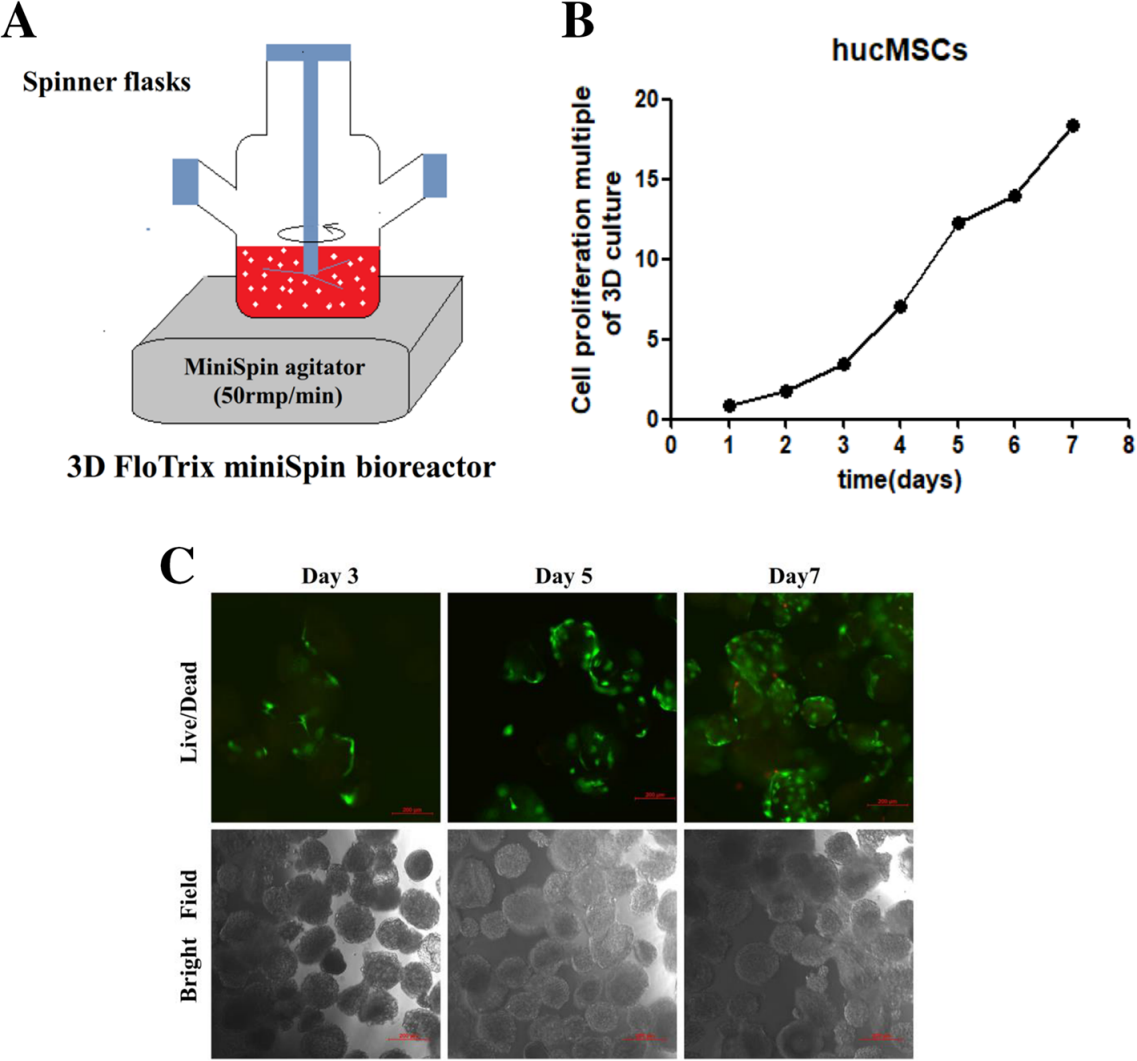
3D cultivation of hucMSCs. **a** 3D dynamic culture system of hucMSCs. **b** Proliferation multiple of hucMSCs cultured in suspension over a period of 7 days. **c** Representative image for hucMSC spheroids on day 3, day 5, and day 7 (green: live cells, red: dead cells). Bar = 200 μm

**Fig. 2:**
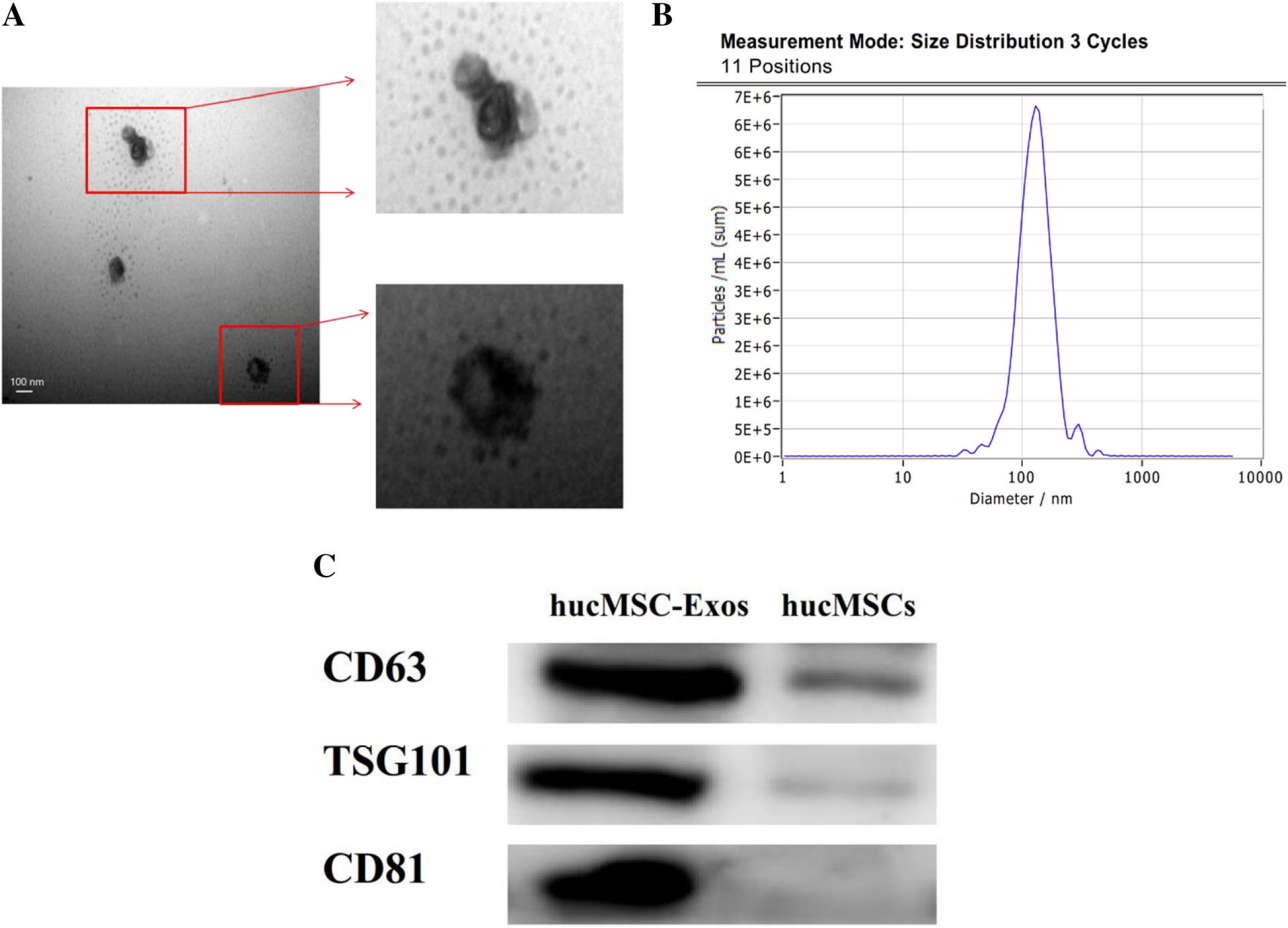
Identification and characterization of hucMSC-Exos. **a** Characteristics of hucMSC-Exos were measured by TEM. **b** The size of average particles displayed 123.9 nm by NTA. **c** The expression of CD81, CD63, and TSG101 was detected in hucMSC-Exos and hucMSCs by Western blotting

To expand the growth of hucMSCs continuously, we used the microcarrier-based 3D dynamic culturing method to increase the surface area available to hucMSCs (Fig. 1a). The number of hucMSCs continuously increased for 7 days (Fig. 2a), and hucMSCs expanded 18.38-fold on the 7th day (Fig. 1b, c).

### Identification and characterization of hucMSC-Exos

Transmission electron microscopy (TEM) analysis revealed that the cup-shaped hucMSC-Exos resembled a double concave disc (Fig. 2a). NanoSight analysis (NTA) showed that the average particle size of hucMSC-Exos was 123.9 nm (Fig. 2b). Western blotting results indicated that hucMSC-Exos significantly expressed the CD63, CD81, and TSG101 marker proteins, but the above protein is not expressed in hucMSCs (Fig. 2c). These findings indicated that hucMSC-Exos were successfully extracted from hucMSCs.

### Tracking hucMSC-Exos in vivo

**Fig. 3:**
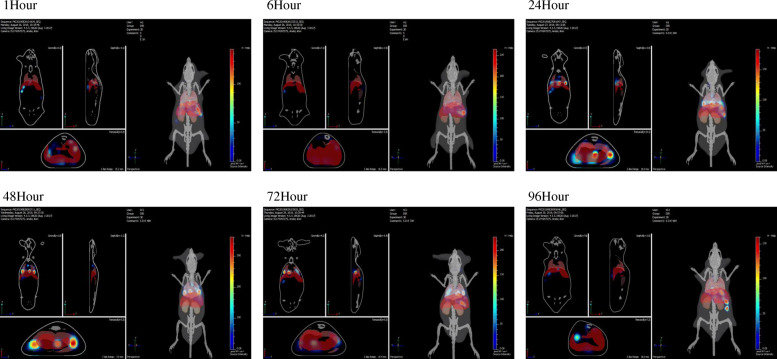
Tracking hucMSC-Exos in vivo. Assessment of bioluminescence imaging signals evaluates whether hucMSC-Exos can migrate to the lung. Representative bioluminescence imaging of animals injected with fluorescent-labeled hucMSC-Exos. The fluorescent signals (DiR: excitation = 750 nm; emission = 780 nm) in the body were captured at different time points (1 h, 6 h, 24 h, 48 h, 72 h, and 96 h) by imaging system Carestream FX Pro

To evaluate the contribution and metabolic processes of exosomes in antagonizing silicosis, we monitored the migration and effect of hucMSC-Exo-labeled-DiR fluorescent dye injected via the tail vein after silica exposure. Bioluminescence imaging showed (Fig. 3) weak fluorescence in the lung after 1 h of tail vein injection. The fluorescence intensity increased gradually over time, reaching a maximum value at 24–48 h, and did not disappear until 96 h. Therefore, these results suggested that hucMSC-Exos could reach the damaged lung tissue, and regular injections of exosomes every 4 days might ensure the continuous presence of exosomes in vivo.

### hucMSC-Exos inhibited silica-induced PF

**Fig. 4:**
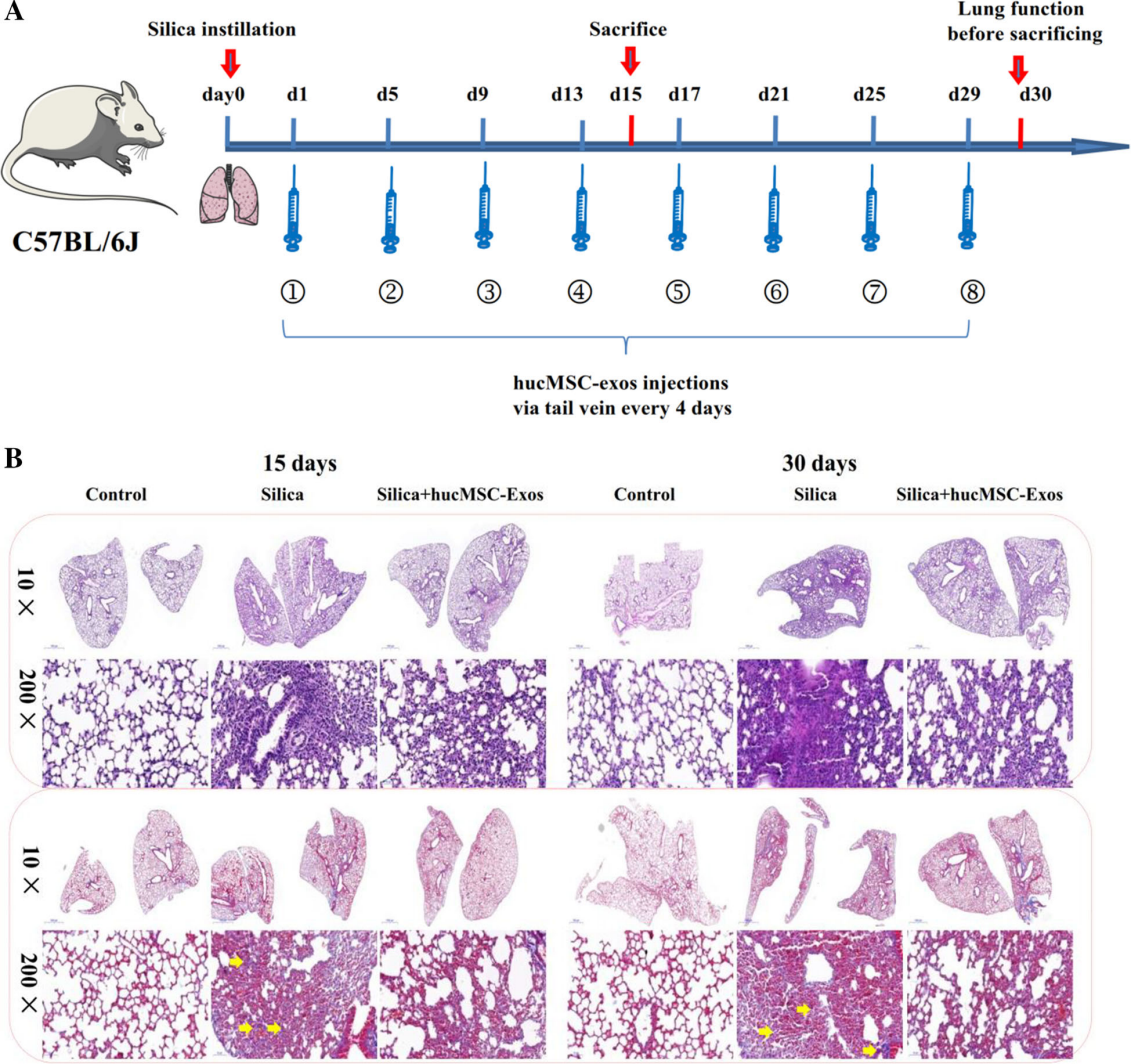
hucMSC-Exos inhibited pulmonary fibrosis in mice (*n* = 3). **a** The experimental process of mice instilled with silica and following hucMSC-Exo treatment. **b** H&E and Masson staining in the lungs of mice in each group at 15 and 30 days (light micrograph magnifications of × 10 and × 200); yellow arrows show typical Masson-positive collagenous fibers. **c** The percentage of Masson staining-positive collagenous fiber was significantly increased in the silica group but decreased in the silica + hucMSC-Exo group at 15 and 30 days mice. **d** The content of HYP was significantly increased in the silica group but decreased in the silica + hucMSC-Exos at different times. The data are presented as means ± SD (*n* = 3). *P < 0.05 compared with the control group. **e**–**j** The expression of collagen deposition marker of COL1A1 and FN was detected by Western blotting and qPCR in mice at each group. *P < 0.05 compared with the control group, ^#^*P* < 0.05 compared with the silica group

To investigate the inhibitory effects of hucMSC-Exos on silicosis fibrosis, we performed a study to compare the effects of silica-induced PF sacrificed 15 and 30 days post the treatments (Fig. 4a). The hematoxylin and eosin (H&E) and Masson staining results showed that at these two time points, the degree of interstitial lung fibrosis, numbers of cell nodules, total lung collagen concentration, and blue collagen deposition were significantly higher in the silica group than in the control group (Fig. 4b, c). The hydroxyproline concentration in the lung presented a marked increase following silica treatment, compared to the case for the control group. However, hucMSC-Exo treatment inhibited these effects (Fig. 4d) in the mice sacrificed 15 and 30 days after the treatment. Western blotting and qPCR analyses showed that the protein and gene expression levels of COL1A1 and FN were upregulated at two time points in the silica group. However, the values of these indices of silica-induced PF were attenuated by the treatment with the hucMSC-Exos (Fig. 4e–j). Our results indicated that hucMSC-Exos could reduce silica-induced PF.

### hucMSC-Exos ameliorated silica-induced respiratory function damage in mice

**Fig. 5:**
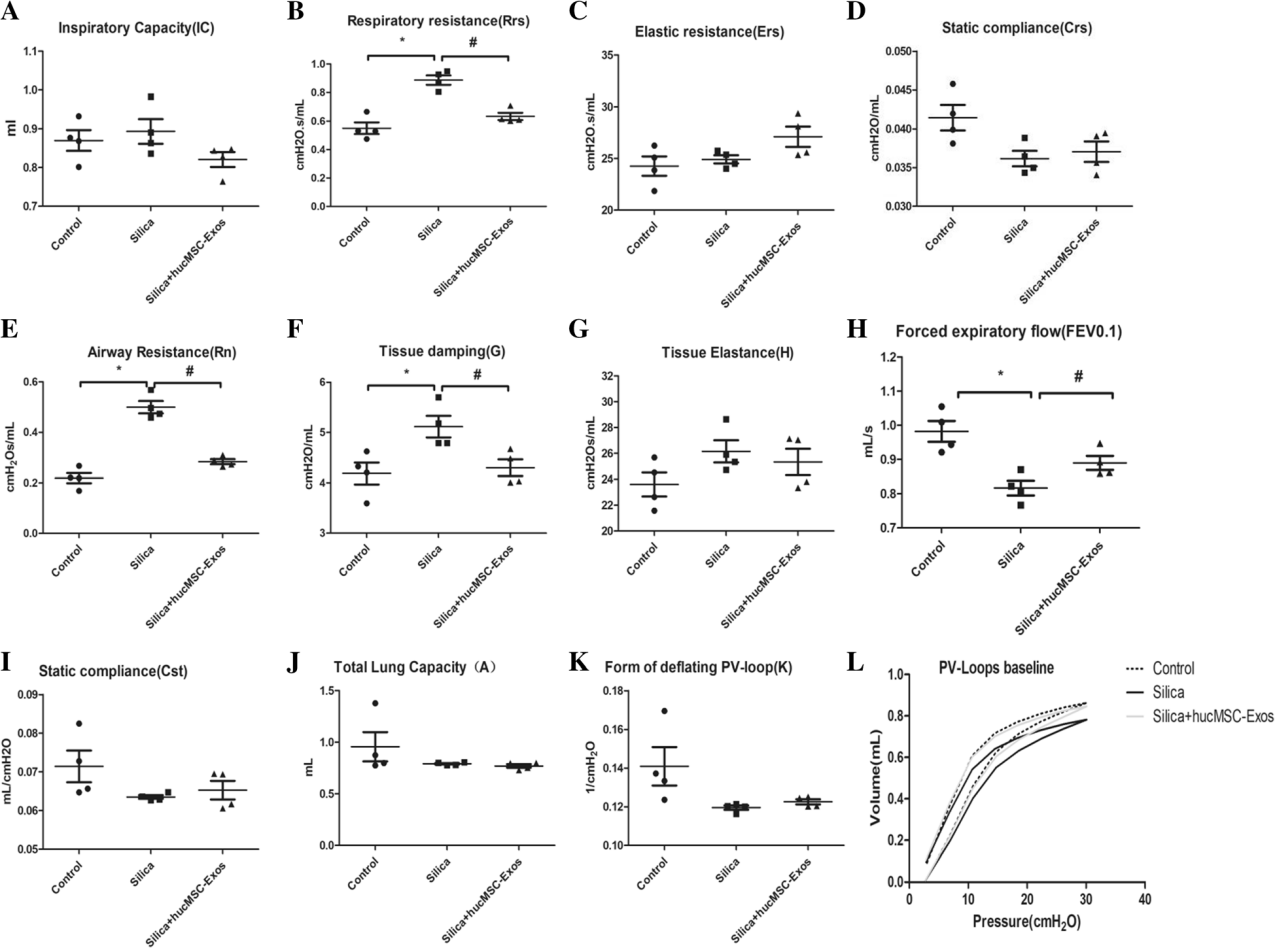
hucMSC-Exos improved the respiratory function damaged by silica in mice (*n* = 4). The flexiVent FX system was used to test the lung function, and results were compared with the reference value. **a** Inspiratory capacity was performed by the model of Deep Inflation. **b**–**d** Respiratory resistance, elastic resistance, and static compliance were performed by the model of Snap Shot. **e**–**g** Airway resistance, tissue damping, and tissue elastance were measured by the Prime wave. **h** FEV model was applied to measure the FEV0.1. **i**–**k** PV loops were used to assess static compliance, total lung capacity, and form of deflating PV loop. PV loop baseline was measured by the PV loops. This was separately calculated for each group. The data are presented as means ± SD (*n* = 4). *P < 0.05 compared with the control group, ^#^*P* < 0.05 compared with the silica group

We assessed the effect of silica and hucMSC-Exos on lung function through forced oscillation technique (FOT), in addition to using traditional methods to evaluate the progression of respiratory diseases. The flexiVent FX system included five models: the basic model of total lung capacity (TLC), Prime wave, Snap Shot, the pressure-volume (PV) loop, and the FEV (forced expiratory volume) model. These models were applied to measure the lung function of the mice in this study. The Snap Shot model was used to access mechanics of the respiratory system, and the inspiratory capacity, normalized by body weight, appeared to be similar in the three groups of the mice (Fig. 5a). Silica increased the respiratory resistance (Rrs), and hucMSC-Exo treatment suppressed this effect (Fig. 5b), compared to the control group. Yet, there were no statistical differences between the values of elastic resistance (Ers) and static compliance (Crs) (Fig. 5c, d).

The Prime wave module was applied to distinguish airway resistance and lung resistance. Silica instillation increased the central airway resistance (Rn) and tissue damping (G); however, these effects declined in the silica + hucMSC-Exo group (Fig. 5e, f), compared to the corresponding reference values. However, there was no statistical significance between the tissue elastance in these two groups.

The PV loop module was estimated via the measurements of total lung capacity (A), static compliance (Cst), form of deflating PV loop (K), and PV loop baseline (Fig. 5i–l). It was an effective way to differentiate fibrosis from other forms of lung damage rapidly. Notably, the PV loops of mice in the silica group showed a downward trend, but there was no statistical significance among the groups (Fig. 5i–l).

The FEV model was applied to measure the FEV0.1 (forced expiratory volume in the first 0.1 s) and FVC (forced vital capacity); this model inflated the mouse lungs to a given pressure and connected the animal’s airways to a negative pressure reservoir, allowing the triggering of a forced expiratory maneuver. Silica instillation decreased the FEV0.1, and hucMSC-Exo treatment increased this parameter (Fig. 5h), compared to the reference values. Taken together, the analysis of respiratory mechanical parameters confirmed that silica induced damage to respiratory function and that hucMSC-Exos could attenuate lung damage in mice.

### Tracking hucMSC-Exos in vitro

**Fig. 6:**
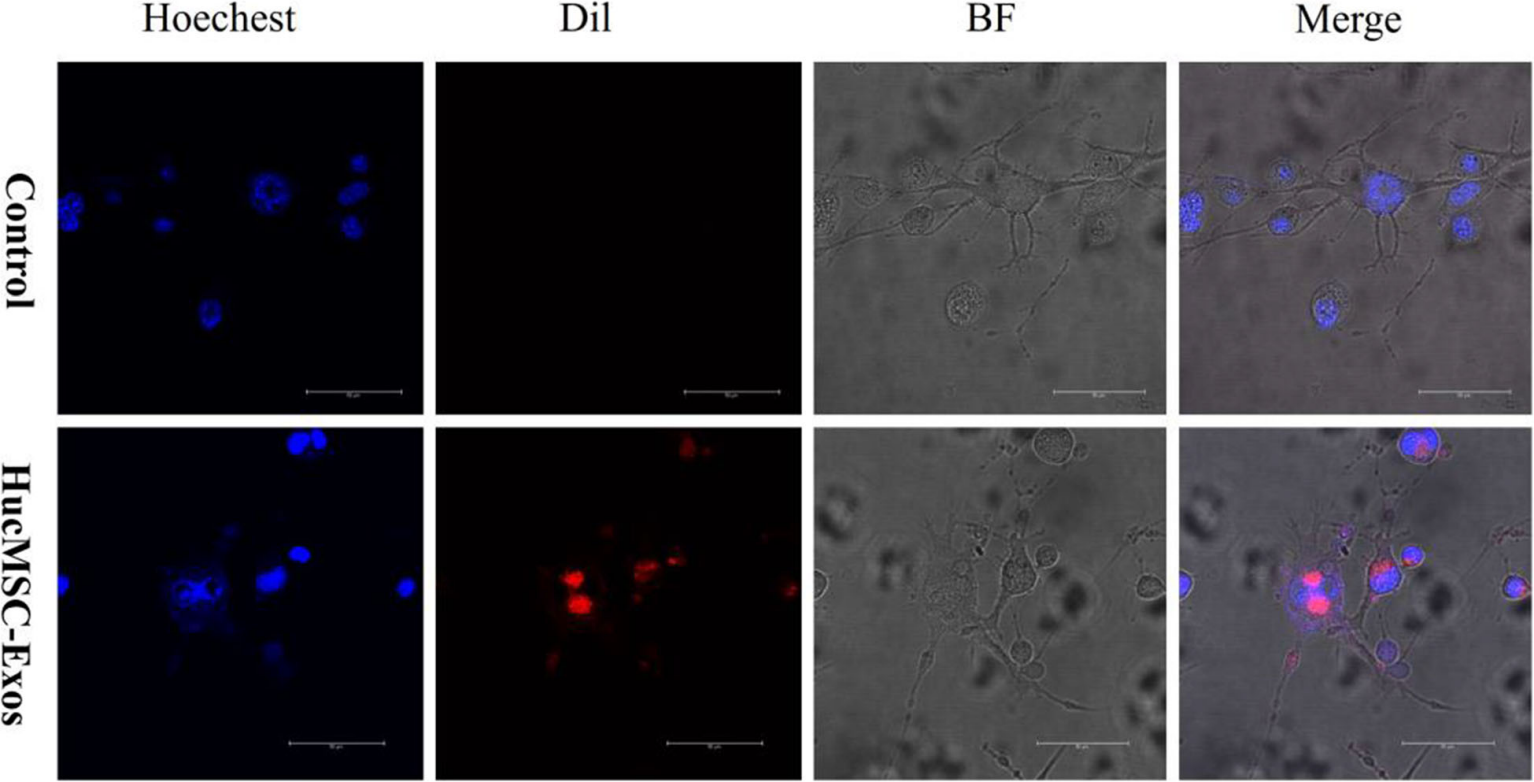
Tracking hucMSC-Exos in vitro. Dil red fluorescent dye (Dil: excitation = 549 nm; emission = 565 nm) was used to label the hucMSC-Exos. Hoechest blue fluorescent dye was applied to label the cell nucleus. BF, bright field

hucMSC-Exos can be located in the tissues only through in vivo tracking, and it is difficult to observe their uptake by cells. Therefore, to ascertain whether hucMSC-Exos were endocytosed by NIH-3T3 cells accurately, we labeled them to enable their tracking in vitro. Dil red fluorescent dye was used to label the hucMSC-Exos. Hoechest blue fluorescent dye was applied to label the cell nucleus. In the in vitro tracking experiment, the labeled hucMSC-Exos were incubated with NIH-3T3 cells, and the cellular uptake of hucMSC-Exos was evaluated via fluorescence microscopy (Fig. 6). The results demonstrated that hucMSC-Exos could enter the cytoplasm of NIH-3T3 cells, and mainly localized to the perinuclear region, implying that the hucMSC-Exos can be internalized by NIH-3T3 cells.

### hucMSC-Exos reduced collagen deposition in NIH-3T3 cells

**Fig. 7:**
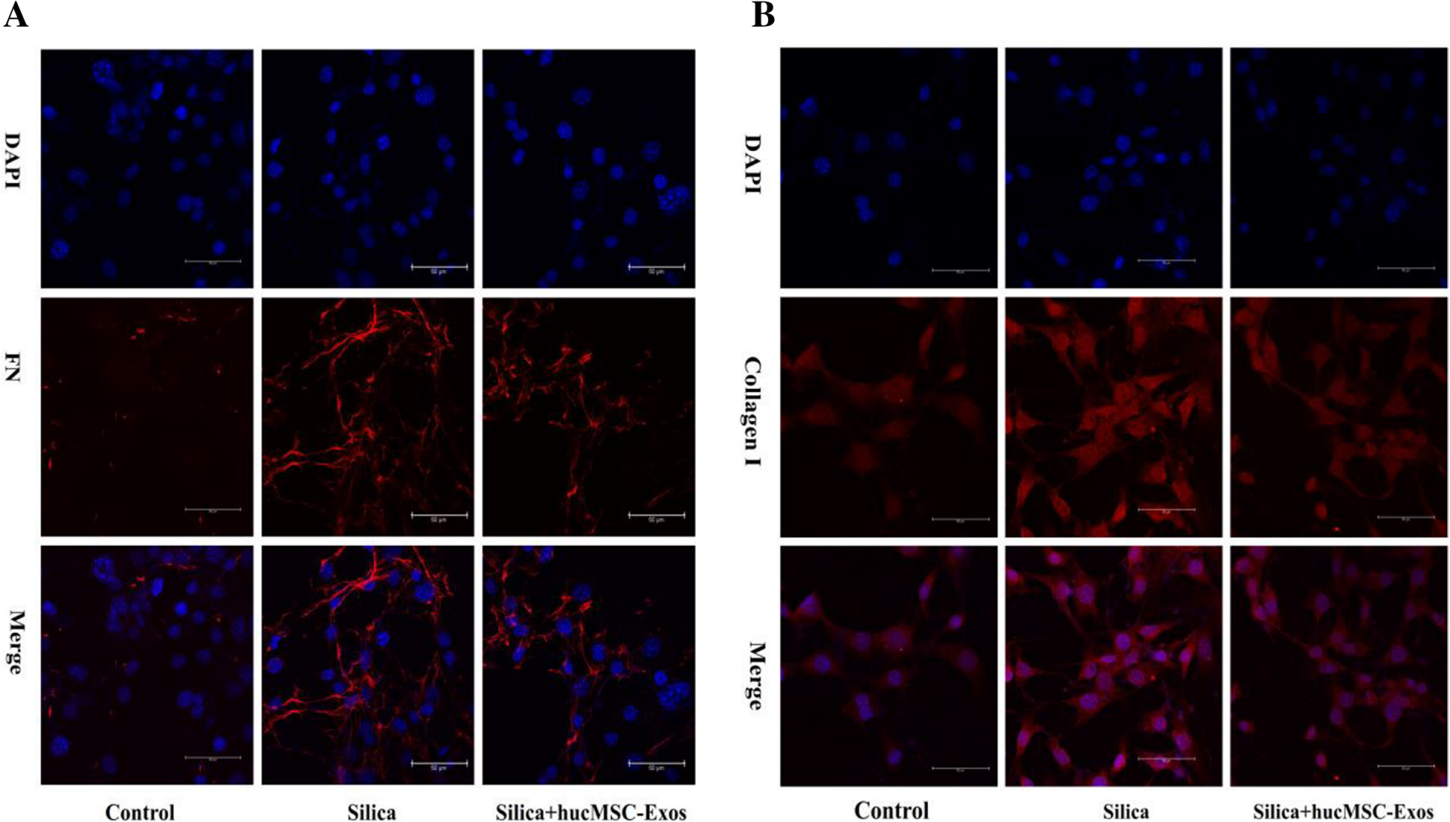
hucMSC-Exos reduced collagen deposition in NIH-3T3 cells (*n* = 3). **a**–**d** Immunofluorescence staining for COL1A1 (red) and FN (red) and semi-quantitative analysis confirmed that silica induced collagen deposition in NIH-3T3 cells. **e**–**g** Detection of COL1A1 and FN expression in hucMSC-Exos by Western blotting and qPCR in NIH-3T3 cells (*n* = 3). *P < 0.05 compared with the control group, ^#^*P* < 0.05 compared with the silica group

The effects of hucMSC-Exos on silica-induced changes of collagen secretion in NIH-3T3 cells were explored. Silica-induced collagen deposition was observed in the NIH-3T3 cells. Figure 7e–g showed that silica induced the upregulation of COL1A1 and FN in the NIH-3T3 cells from the silica group, compared to the case for those from the control group, as ascertained using Western blotting and qPCR analyses (*P* < 0.05, Fig. 7d). Meanwhile, the results revealed that silica induced changes in collagen deposition of NIH-3T3 cells and that this change was attenuated following the treatment with the hucMSC-Exos.

This study also assessed the expression of collagen deposition markers using immunofluorescence staining. Semi-quantitative results showed that the expression of COL1A1 and FN increased in the silica group, relative to that in the control group (Fig. 7a–d). Meanwhile, this change declined following the treatment with the hucMSC-Exos.

## Discussion

Previous studies have shown the beneficial effects of hucMSC-Exo administration in experimental models of fibrosis, such as radiation-induced lung fibrosis [16, 17] and CCl4-induced liver fibrosis [18]. However, few studies have employed hucMSC-Exos as a therapeutic strategy for silica-induced PF. In order to address this question, we developed a 3D dynamic system for culturing hucMSC spheroids in a microcarrier suspension to yield exosomes from serum-free media and performed experiments in which C57BL/6J mice were treated with hucMSC-Exos. We observed that hucMSC-Exos could antagonize silica-induced PF and modulate pulmonary function. These results revealed that hucMSC-Exos decreased collagen deposition in NIH-3T3 cells exposed to silica. These findings provide new insights and methods for the treatment of silica-induced PF.

Some studies [19, 20] have reported a 3D culture system for hucMSCs to yield exosomes. Haraszti et al. [19] confirmed that 3D cultures yield 20-fold more exosomes (3D hucMSC-Exos) than 2D cultures (2D hucMSC-Exos), and 3D hucMSC-Exos are seven times more efficient in small interfering RNA transfer in terms of targeting organs, as compared to 2D hucMSC-Exos. In this study, we employed a novel method for 3D dynamic cultures of hucMSCs to continuously yield exosomes. hucMSCs were grown on the surfaces of microcarriers, which were distributed in serum-free medium by rotating wall vessel bioreactor and stirring in a spinner flask. This method can overcome many limitations associated with 2D culture systems. First, the dynamic conditions may aid in the movement of nutrients toward the spheroids and waste products away from the spheroids, facilitating the viability of cells, thus yielding more hucMSC-Exos than in the 2D culture within the same culture volume [21]. Second, stem cells cultured in 2D are prone to aging owing to the number of passages, whereas a 3D culture can avoid cell aging and ensure the quality of exosomes. Finally, this method can recapitulate the native 3D cellular microenvironment in vivo and maintain hucMSC stemness, which guarantees the function of exosomes [18].

Cell-free therapy based on exosomes represents an attractive approach because of its advantages with respect to high stability, non-immune rejection, ease of reaching the wound tissues, and free of vascular obstructive concern for tissue engineering [22]. The size of exosomes ranges from 40 to 150 nm [11]. In our study, exosomes comprised particles less than 150 nm as assessed with TEM and NTA, yielding from 3D hucMSCs. Some markers of exosomes were detected with Western blotting, such as CD81, TSG101, and CD63. These results confirmed that exosomes had been successfully extracted from the cell supernatant, and were applied to our subsequent research.

The current work traced the distribution of hucMSC-Exos in lung tissues at different time points and found that DiR-labeled hucMSC-Exos accumulated in lung tissues after tail intravenous injection of 1 h. Although hucMSC-Exos were still present on the 4th day after injection, this density had been reduced significantly, and the signal disappeared on the 5th day after injection. The tracking results in vitro showed that hucMSC-Exos could enter the cytoplasm of fibroblasts. Similar observations have been reported in other cell types previously. Srivastava et al. co-cultured dye-labeled exosomes and tumor cells, which confirmed that the exosomes entered into the cell cytoplasm physically [23]. This phenomenon might be similar to the homing abilities of stem cells. The cell membranes of stem cells invaginated into the cytoplasm, covering miRNAs and proteins and forming multivesicular endosomes, which combined with the cell membranes to release exosomes to the surrounding environment [24]. Based on the surface receptors and adhesion molecules of stem cells [25, 26], exosomes presented similar homing abilities like their parent cells and could be recruited to the wound region. In our previous study, we found that stem cells could lead to homing lungs in the treatment of silica-induced PF [27]. It has been postulated that exosomes could maintain the integrity of the cytomembrane to avoid degradation, and avoid detection by the immune system. However, the specific mechanism that exosomes reach the lung tissue of homing ability requires further investigation.

In this study, our results showed that fibrosis could be reduced with the repeated injections of hucMSC-Exos in mice. It is worth noting that the mice exposed to silica were injected 200 μg hucMSC-Exos at day 1 post-silica instillation, and hucMSC-Exos were injected every 4 days. Once injected, hucMSC-Exos mainly accumulate in the lungs and are able to repair lung injury successfully and thus can be considered a treatment approach. Specifically, when mice were treated with hucMSC-Exos, there was a significant amelioration of silica-induced PF in 15 days and 30 days. In particular, Dinh et al. [28] have utilized lung spheroid cell-exosomes (LSC-Exos) to treat silica-induced lung fibrosis for seven consecutive days by decreasing both collagen accumulation, which is consistent with our results surrounding the apparent impact of hucMSC-Exos administered on silica-induced PF. Additionally, in vitro, these results revealed that hucMSC-Exos decreased silica-induced collagen deposition, including COL1A1 in NIH-3T3 cells as well as in lung tissue from mice exposed to silica. Therefore, we believe that hucMSC-Exos respond to their microenvironment by releasing proteins or miRNAs [9], and the contents in an inflammatory environment would contain inhibiting lung fibrosis properties. The evidence described indicates the therapeutic potential of exosomes for silica-induced PF. Based on these studies, we will further explore the potential mechanisms of exosomes against pulmonary fibrosis.

We carried out lung function measurements to further evaluate the changes of pulmonary function-in silica-induced PF in mice, along with the protective effects of hucMSC-Exos by FOT measurements objectively. FOT measurements are an invasive method that can measure relevant parameters physiologically and describe the mechanical properties of the respiratory system accurately, which have been used to explore pathophysiological changes associated with fibrosis in mice models of respiratory diseases [29, 30]. The classical parameters of respiratory mechanics, resistance, and compliance are related to the resistance of air in and out of the lungs as well as the expandability of the respiratory system. These measurements are based on the analysis of pressure, volume, and flow signals obtained from the oscillating airflow waveform applied to the airway opening of the subject [31, 32]. Exposure to the silica increased respiratory resistance (Rrs), airway resistance (Rn), and tissue damping (G) in mice. In addition, FEV0.1 decrease was induced by silica, and the PV loop showed a significant downward shift, indicating that silica reduced the elastic recoil of the lung. Devos et al. reported an increase in airway resistance (Rn) and tissue damping (G), but FEV0.1 decreased in mice with bleomycin-induced pulmonary fibrosis [33, 34]. However, other parameters of pulmonary function, such as inspiratory capacity (IC) and static compliance (Cst), were almost unchanged in the silica group compared to the normal mice, indicating that the volume of the lung was slightly changed at the early stage of silica-induced PF. This is consistent with the phenomenon that silica-induced PF may appear at an early stage without symptoms. hucMSC-Exos caused an upward shift in the PV loop, suggesting an increase in elastic recoil. At the same time, hucMSC-Exos also alleviated respiratory resistance (Rrs), airway resistance (Rn), and tissue damping (G), indicating hucMSC-Exos have a therapeutic effect on both tissues and airways. Overall, the results of the lung functional measurements confirmed that hucMSC-Exos could blunt lung impairment induced by silica.

In conclusion, this study discovered that exosomes from 3D-cultured hucMSCs had the potential to inhibit silica-induced PF and improve lung function. The research revealed that hucMSC-Exos decreased collagen deposition in NIH-3T3 cells exposed to silica. Furthermore, future studies should validate the mechanism directly whether miRNAs and proteins are enriched for this application. Ultimately, these findings suggest that hucMSC-Exos could be a promising therapeutic strategy for inhibiting silica-induced PF.

## Supplementary Information

**Additional file 1.**

## Data Availability

All data and materials are available in the manuscript.

## Abbreviations

PF: Pulmonary fibrosis
3D: Three-dimensional
hucMSC: Human umbilical cord mesenchymal stem cell
MSCs: Mesenchymal stem cells
TEM: Transmission electron microscopy
NTA: NanoSight analysis
HYP: Hydroxyproline
