# Supplementary material for: Exosomes derived from three-dimensional cultured human umbilical cord mesenchymal stem cells ameliorate pulmonary fibrosis in a mouse silicosis model
Source: Stem Cell Res Ther. 2020 Nov 25;11:503. doi: 10.1186/s13287-020-02023-9 (PMC7687745; doi:10.1186/s13287-020-02023-9)
Supplement: Supplementary file 1 — Additional file 1. [file 13287_2020_2023_MOESM1_ESM.docx]

**Online method**

**Cell culture**

HucMSCs and media were purchased from NUWACELL Co. Ltd. (Hehui, China). Eligibility criteria of HucMSCs was tested, searching for website for details (<http://www.nuwacell.com/index.php>?c= content&a=show&id=347). The cells were cultured in basal medium supplemented with 4% supplement medium, a serum-free medium that was used to ensure exosome secretion from cells. Cells between passages 3 and 5 were used for experiments. 3D FloTrix miniSpin bioreactor (Beijing CytoNiche Biotechnology Co. Ltd., Beijing, China) is used for scalable microcarrier-based 3D dynamic culture of hucMSCs, and it mainly includes a miniSpin agitator and spinner flasks of three volumes. Spinner flasks (250 mL) containing 320 mg (5×10^4^ cells/mL) of 3D TableTrix Microcarriers (Cytoniche Biotech. Ltd. Beijing, China) were autoclaved. HucMSCs were seeded to a density of 5×10^4^ cells/mL in basal medium supplemented with 4% supplement medium; the speed of the miniSpin agitator was set to 50 rpm/min, and cells were cultured at 37 °C in a 5% CO_2_ incubator. Cells were then homogenously spread on microcarriers, 250 mL of serum-free medium was added within seven days, and the medium was replaced with fresh medium every second day. During the cell culture, cell viability was determined using calcein AM and propidium iodide (PI) kit (Wako,Japan) according to manufacturers’ instruction.

Cell-laden microcarriers were allowed to sediment to the bottom of the sampling tube/EP tube, and medium was aspirated carefully to ensure that microcarriers were not removed. [Subsequently](file:///C:\Program%20Files%20(x86)\Youdao\Dict\7.5.1.0\resultui\dict\?keyword=subsequently), 3D FloTrix™ Digest (CNR001-500, CytoNiche Biotech, China) were added at a ratio of 0.15 mL/mg microcarrier and incubated at 37°C for 30 min. When harvested in culture vessels, gentle pipetting was performed for sampling tubes/EP tubes every 10 min to assist dissolution of microcarriers and dissociating cells. Cells number and viability were counted by Trypan Blue exclusion assay using an automatic cell counter (Countstar Biotech, ALIT Life Science, China).

RAW264.7 and NIH-3T3 cells were seeded in DMEM (HyClone, Beijing, China) supplemented with 10% fetal bovine serum (FBS), and incubated in a 5% CO2 incubator at 37°C. When the RAW264.7 cells reached 80% confluence, they were incubated with 50 μg/mL silica for 24 h. After RAW264.7 cells were treated with 50μg/mL silica for 24 h, the supernatant was collected. NIH-3T3 cells were maintained with the original silica supernatant for 24 h and harvested for future experiments.

**Isolation and identification of hucMSC-Exos**

Serum-free medium of the 3D culture was centrifuged at 3000 g for 15 min to remove dead cells and cellular debris, and the culture medium containing exosomes was retrieved. The recovered supernatant was filtered with a 0.22 µm filter (Millipore, Billerica, USA), and 15 mL of the supernatant was added to the Amicon Ultra-15 centrifugal filter unit (100 kDa; Millipore) and centrifuged at 4000 g to 1 mL. The ultrafiltration liquid was washed twice with PBS, and ultrafiltered again at 4000 g to 1 mL. One-fifth volume of Exoquick exosome precipitation solution (System Biosciences, Palo Alto, USA) was added to the ultrafiltrate, mixing thoroughly by inversion. After incubation for 12 h, the mixture was centrifuged at 1500 g for 30 minutes, and the supernatant was removed by suction. Resuspend the exosomal pellet in 500 μL PBS. All steps are performed at 4ºC. The BCA protein analysis kit (Boster, Wuhan, China) was used to determine the protein content of exosomes.

**Animal model of silicosis and design**

A total of 60 male C57BL/6J mice were purchased from Vital River Laboratory Animal Technology (Beijing, China) weighing 20-22 g. The mice were kept in a temperature-controlled room (24°C±1°C) with a 12:12-h light: dark cycle, and sufficient food and water were provided. The mice were anesthetized with 350 mg/kg of tribromoethanol (Sigma, St. Louis, USA). Subsequently, the mice were treated with 0.1 mL of intratracheal silica suspension (2.5 mg), except for the control group, which received the same volume of saline. To investigate the effects of hucMSC-Exos *in vivo*, the mice were administered hucMSC-Exos via tail vein injection every four days at a dose of 200 μg/100 μL. Furthermore, hucMSC-Exo treatment was performed at day 1post silica instillation. The mice in the control and silica groups were injected with an equal volume of saline; the mice in these groups were sacrificed on the 15th and 30th day respectively. The lung function of the mice was evaluated before sacrifice, and lung tissue sections were collected for examination.

***In vivo* tracking**

Mice were intravenously injected with hucMSC-Exo labeled with fluorescent DiR and anesthetized with tribromoethanol 350 mg/kg for 5-10 minutes. The Carestream FX Pro imaging system (Bruker BioSpin MRI GmbH, Ettlingen, Germany) was used to capture the fluorescent signal at different time points (1, 6, 24, 48, 72, and 96 h) in the body. In order to determine the anatomical orientation, a white light/gray-scale picture was created and used with the fluorescent signal (DiR: excitation =750nm; emission=780nm). When organs containing hucMSC-Exos-DiR were observed by software visualization (Bruker BioSpin MRI, Ettlingen, Germany), the distribution and intensity of hucMSC-Exos with DiR were measured to reflect the location and metabolism conditions.

**Lung function measurements**

The flexiVent FX system (SCIREQ, Inc., Montreal, Canada) was used for respiratory function measurement. The system was equipped with an FX2 module and operated using the Flexi Ware v8.0 software. The mice were anesthetized with pentobarbital (50 mg/kg, CEVA, Brussels, Belgium) by intraperitoneal injection. The trachea was exposed so that a cannula could be inserted, which was connected to the tubing system extending from the machine. The mice were examined at a frequency of 150 breaths/ min, and lung function was measured automatically. Briefly, the inspiratory capacity was measured by lung inflation to 30 cm H_2_O over 3 s. The values of overall respiratory system resistance (Rrs), compliance (Crs), and elastance (Ers) were obtained from single-frequency forced oscillation maneuvers. The model of the prime wave maneuver was utilized to calculate the respiratory input impedance. The parameters of airway resistance (Rn), tissue damping resistance (G), and tissue elasticity (H) were analyzed, and the coefficient of determination was ≥0.95. PV curves were determined, and step-wise data for increased and decreased airway pressures were calculated. Static C (Cst) was calculated from the slope of each curve. The area (hysteresis) of the PV curve and shape parameter (A or K) describing the deflation limb of the PV loop were also calculated. The FEV model was applied to measure FEV0.1 and FVC, which enabled the triggering of a forced expiratory maneuver via inflation of mouse lungs to a given pressure followed by connection of the animal’s airways to a negative pressure reservoir.

**Western blotting**

The samples of hucMSC-Exos were subjected to SDS-PAGE on 10% gels, followed by Western blotting. Proteins were blotted on polyvinylidene difluoride (PVDF) membranes, which were then blocked with 5% milk in PBS/0.1% Tween 20 (PBS/Tween). The following primary antibodies were used: CD81 (1:1000) and TSG101 (1:1000) were purchased from Abcam (Abcam, USA) and CD63 (1:1000) was procured from Proteintech (Proteintech, USA). The blots were visualized using horse radish peroxidase (HRP)-conjugated secondary antibodies and the ECL Detection Reagent (Absin, Shanghai, China), and were imaged by Tanon-5200 system (Beijing Yuan Ping Hao Biotech, China).

After 24 h of incubation, the lysis buffer from the KGP2100 kit (KeyGen Biotech, Nanjing, China) was added to NIH-3T3 cells, and the protein was extracted according to the manufacturer’s instructions. The protein concentration in each well was measured by the BCA protein assay kit (Boster, Wuhan, China). Briefly, 20 μg of protein was resolved on 8% SDS polyacrylamide gels and transferred onto PVDF membranes (Millipore, Billerica, USA), which were blocked with 5% BSA solution and incubated with primary antibodies overnight at 4 °C. The primary antibodies, COL1A1 (1:100) and FN (1:200), were purchased from Santa Cruz (Santa Cruz, USA) and GAPDH (1:1000) was obtained from Cell Signaling Technology (Cell Signaling Technology, USA). After washing with TBST thrice (10 min each) at room temperature, the PVDF membranes were incubated with HRP-conjugated secondary antibodies for 90 min; the anti-rabbit or anti-mouse IgG HRP-linked antibody was also obtained from Cell Signaling Technology. The membranes were washed a second time with TBST for 15 min (5 min each). ECL detection reagent (Absin, Shanghai, China) was used to detect the signals. and the signals were imaged by Tanon-5200 system (Beijing Yuan Ping Hao Biotech, China). The quantification was calculated by ImageJ software to analyze the intensity of the gray scale images.

**RNA isolation and reverse transcription quantitative PCR (RT-qPCR)**

Total RNA was extracted from the cells using TransZol Up (ET111, TransGen Biotech, Beijing, China), and mRNA was transcribed to cDNA using the TransScript First-Strand cDNA Synthesis SuperMix (AT301, TransGen Biotech, Beijing, China). RT-qPCR was performed with the CFX96 real-time qPCR detection system (Bio-Rad, Hercules, USA) using the SYBR Green qPCR kit (AQ141, TransGen Biotech, Beijing, China). The levels of gene expression was calculated by normalizing to the glyceraldehyde-3-phosphate dehydrogenase level. The COL1A1 sense sequence was 5′ -GCTCCTCTTAGGGGCCACT-3′, and the antisense sequence was 5′-CCACGTCTCACCATTGGGG-3′. The FN sense sequence was 5′- CTATAGGATTGGAGACACGTGG-3′, and the antisense sequence was 5′- CTGAAGCACTTTGTAGAGCATG-3′.
